# Adolescents’ Responses to a School-Based Prevention Program Promoting Healthy Eating at School

**DOI:** 10.3389/fpubh.2017.00309

**Published:** 2017-11-20

**Authors:** Roel C. J. Hermans, Hanneke de Bruin, Junilla K. Larsen, Fréderike Mensink, Annet C. Hoek

**Affiliations:** ^1^Behavioural Science Institute, Radboud University, Nijmegen, Netherlands; ^2^The Netherlands Nutrition Centre, The Hague, Netherlands; ^3^To Remind You, Eindhoven, Netherlands; ^4^BehaviourWorks Australia, Monash Sustainability Institute, Monash University, Melbourne, VIC, Australia

**Keywords:** mixed-methods research, eating behavior, food choice, universal prevention, school canteen

## Abstract

**Background:**

To improve the effectiveness of school-based programs that aim to promote adolescents’ healthy food choices, it is essential to understand the views and behaviors of the target group. This study aimed to get a better understanding of adolescents’ food and health perceptions and their willingness to be involved in a specific school-based prevention program, i.e., the Dutch “*Healthy School Canteen Program*.”

**Methods:**

This study used a mixed-methods research design. First, seven semi-structured focus groups were conducted using a selective sample of 42 Dutch adolescents (25 girls, 17 boys, aged 13–16 years). Second, an online survey among 133 adolescent respondents (72 girls, 61 boys, aged 12–19 years) using snowball sampling was conducted. Content analysis was performed to make inferences about the focus group discussions, whereas statistical analyses were conducted to analyze the survey data.

**Results:**

Findings from the group discussions indicated that healthy eating was only an issue of importance when adolescents perceived negative physical changes (e.g., with regard to looks or physical performance). Adolescents also indicated that they clearly wanted to make their own food and beverage choices at school. The quantitative data indicated that taste, price, and variety were seen as the most important aspects of a healthy food assortment (mean scores 8.1, 7.8, and 7.7 on a 10-point scale, respectively). In general, a majority of the adolescents (64%) expressed that students should be involved in the organization of a healthy food environment in schools. At the same time, however, adolescents were not willing to participate themselves. This was mostly because they were skeptical about their ideas being heard and put into action by their schools.

**Conclusion:**

School-based prevention programs, such as the *Healthy School Program*, should take into account that adolescents have a low risk perception of unhealthy eating and are seeking food choice autonomy. In addition, schools should not lose sight of product price, taste, and variety to make their food assortment attractive to students. If schools aim to involve adolescents in prevention programs that promote healthy eating, it is essential that they have a formal student involvement process that ensures that adolescents’ suggestions are valued.

## Introduction

The prevalence of overweight (including obesity) in children and adolescents in the WHO European region is alarming. Recent figures indicate that 1 in 3 11-year olds is overweight or obese (1). Although the prevalence of pediatric obesity is less profound in The Netherlands than in most other European countries, still 18% of teenagers between 12 and 17 years are overweight or obese (2). Healthy eating is key to good health as well as maintaining a healthy weight. Unfortunately, many European and Northern-American children and adolescents have an unhealthy food pattern with an excessive intake of added sugar or fat and inadequate intakes of fruits, vegetables, and whole grains making them at risk for (further) weight gain [e.g., Ref. (3–5)]. By now, there is ample evidence that the current obesogenic environment, in which energy-dense food products are cheap, easily available, and offered in large portion sizes, exerts a large influence on individuals’ food choices and consumption (6, 7).

Given that adolescents spend a considerable proportion of their time at school (8, 9), the school context offers an effective and efficient way to reach a large segment of the young population (10). A potentially viable strategy to teach young people about nutrition and to provide them with skills to make healthy food choices is nutrition education (11). Given the influence the environment can exert on young people’s food choices, an additional strategy is to create a healthy school food environment that facilitates children to choose healthy food products (12). Indeed, it has been found that a healthy school food environment could positively influence youngsters’ diet or food purchases at school [e.g., Ref. (13, 14)]. Although the school food environment has received increased attention in recent years, there is still a lot to improve when it comes to the foods and drinks available at schools. In many Western countries, unhealthy foods and drinks containing excessive sugar, salt, and saturated fat are still widely available [e.g., Res. (15–17)]. In The Netherlands, for instance, the food assortment in vending machines is often less favorable compared with nutritional guidelines (18, 19).

To support Dutch secondary schools and schools for vocational training with improving their school food environment, The Netherlands Nutrition Centre has developed the *Healthy School Canteen Program*. In The Netherlands, there is no compulsory system of school meals. Instead, students may choose to bring their own packed lunches (e.g., sandwiches) or to purchase supplementary food items at the canteen and vending machines at school. *The Healthy School Canteen Program* aims to support schools in making their canteen’s offering healthier. The program consists of a four-step roadmap for schools, which starts with an “Inventory of current cafeteria offerings, curriculum, and policies,” followed by an “Action Plan,” an “Implementation Phase” and, an “Evaluation Phase.” While completing these four steps, schools are guided toward a healthy school canteen; a canteen in which between 60% (“Silver Award”) to 80% (“Gold Award”) of the visible assortment meets the Guidelines of The Netherlands Nutrition Centre (20).^1^ In 2015, between 30 and 50% of Dutch secondary schools and between 20 and 70% of Dutch vocational schools started to implement *The Healthy School Canteen Program* (21). Ultimately, the Dutch government would like to see all school canteens in The Netherlands become healthy school canteens. Therefore, the Ministry of Public Health, Welfare and Sports has assigned The Netherlands Nutrition Centre with assisting schools in making their canteens healthier.

Identifying perceived needs and barriers to a healthy school food environment contributes to adequate program development. Furthermore, the adoption, implementation, and maintenance of school-based programs can be improved by following a bottom up rather than a top down approach only (22). This includes involvement and acceptance by the target population, in this case adolescents (23). *The Healthy School Canteen Program* was originally set out to follow a multicomponent strategy involving all stakeholders: students, teachers, parents, school boards, canteen employees, Municipal Health Services, and caterers (12). Although The Netherlands Nutrition Centre specifically advises all schools to involve their students in *The Healthy School Canteen Program*, schools often report that it is challenging to actually engage adolescents with the program. This is unfortunate because the lack of student involvement may impact the effectiveness of *The Healthy School Canteen Program*, as behavior change is less likely to occur if youngsters do not consider the target behavior to be important or acceptable for themselves (23). A first step to increase adolescents’ involvement in school-based programs that promote healthy eating is to gain a better understanding of adolescents’ food and health perceptions and their eating and buying behaviors within the context of their school.

There have been a number of studies exploring stakeholders’ views on obesity prevention in schools, for example, in the U.S., Switzerland, Belgium, and The Netherlands (17, 24–26), but only a few have focused on adolescents specifically. De Ridder and colleagues, for instance, demonstrated that Dutch adolescents’ risk perception regarding unhealthy eating was low; as long as they felt healthy, they felt no need to change their (eating) behavior (27). Similarly, Stevenson and colleagues found that Irish adolescents viewed healthy eating as an unnatural, unpleasant short-term activity to avoid the stigma of obesity or to enhance physical attractiveness (28).

Also, it was found that Northern Irish adolescents indicated that food price, value for money, taste, and visual appearance were the most important factors influencing their food choice. Further, these adolescents were in favor of rewards-based interventions (e.g., individualistic and immediate rewards when buying healthy foods) (29). Until now, it is unknown whether the same would hold for Dutch adolescents within the context of the *Healthy School Canteen Program*.

The overall aim of this study was to gain a better understanding of adolescents’ food and health perceptions along with their eating and buying behaviors within the context of their school environment. Second, we examined adolescents’ willingness to be involved in the organization of a healthy food and drinks assortment at school. By doing so, this study might provide policy makers and intervention developers with valuable information on how to improve interventions for a healthy food environment and to promote healthy food choices in secondary schools.

## Materials and Methods

### Study Design

To obtain complementary data on food and health perceptions and eating and buying behaviors of adolescents, we adopted a mixed-methods approach (30). Semi-structured focus groups were conducted with adolescents to get a deep understanding of the factors influencing their daily food choices, particularly at school. In addition, adolescents participated in an online survey on their daily eating and buying behavior within the context of their school environment. They also answered questions about their willingness to participate in interventions aimed at improving the food and beverage assortment at schools. This part of the study was conducted to quantify the results of the focus groups with self-reported behavioral questions and ratings. This study was conducted in October/November 2014. Participants received a 5 Euro gift voucher when taking part in the focus group and another 5 Euro voucher when completing the online questionnaire.

### Focus Groups

#### Study Population

A selective sample of seven secondary schools (i.e., general secondary education and preparatory secondary vocational education) in The Netherlands was selected by purposive sampling. The schools were drawn from the researchers’ network, on basis of their geographical region (e.g., rural or urban area from the four corners of The Netherlands). Recruitment was done by the schoolteachers from these schools. Seven focus groups at 7 different schools were conducted with 42 adolescents (17 boys and 25 girls). The sample represented boys and girls from different ages, levels of education, and regional areas (see Table 1). The teachers were asked to select and invite students aged 13–16 years from different educational backgrounds. This resulted in focus groups with a mean group size of six adolescents from the same school. All groups had mixed education levels to feed the discussion from multiple perspectives (31). Although homogeneity with regard to adolescents’ sex is generally recommended for adolescent focus groups (32), it was not possible for this study due to scheduling reasons; four out of seven groups consisted of both boys and girls from the same age range.

**Table 1:** Sample characteristics.

		Focus groups (*N* = 42)	Survey (*N* = 133)
Age (years) mean (range)		14.8 (13–16)	14.8 (12–19)

Gender	Female	60%	54%
	Male	40%	46%

Education level	Low (VMBO)	38%	47%
	Medium (HAVO)	29%	23%
	High (VWO)	33%	30%

Regional area	North	38%	17%
	South	17%	32%
	West	17%	11%
	East	29%	40%

BMI (kg/m^2^) mean (range)		Unknown	20.2 (15.8–28.7)

*VMBO is equal to vocational training level; HAVO is equal to senior secondary general education level; VWO is equal to pre-university education level*.

#### Description of Focus Groups

The focus groups were held at the different schools during school time. The groups lasted on average 75 min, were video taped, and facilitated by a moderator from the research team. All participants signed a consent form immediately preceding the focus group indicating willingness to participate in the group. All participants provided their permission for the video recording of their group session. The moderator used a semi-structured approach with open questions following a discussion guide and verbally summarized the group’s comments after each question. The discussion guide was primarily based on themes previously identified in research on adolescents’ views of food and eating (28) and their perceptions of (healthy) school canteens (29), and their own involvement in organizing a (healthy) school canteen. Thus, in each focus group, a series of questions were asked pertaining to healthy eating (at school), *The Healthy School Canteen Program*, and the involvement of school and students in the organization of a healthy food environment in schools.

The core structure was as follows:

1. Healthy foods and eating behavior: perceptions, practices, and importance attached to food and health.
2. Influences on food choice: the role of others, situations, types of food and drinks, particularly during school hours.
3. School canteen as food provider: perceptions of the current offering and the role of the school canteen in food choice and consumption.
4. Student involvement with the school canteen: current involvement, expected future involvement with *The Healthy School Canteen Program*, and solution areas to promote this.

There was a flexible approach in the order and number of questions asked for each of the four key discussion points, which we tailored to the input and dynamics of the group (33).

#### Analysis

The focus groups were transcribed verbatim by the first author and a research assistant. The transcripts were analyzed manually by using a coding scheme based on the key topics from the discussion guide. This was further refined using an inductive approach as specific themes emerged from the data itself. During the process, the key findings and themes were mutually discussed and agreed upon with the second and last author, who also read the full transcripts.

### Survey

#### Study Population

The survey was completed by 133 adolescents (61 boys and 72 girls) of which 34 also participated in the focus groups. The sample characteristics are depicted in Table 1. A snowball sampling strategy was used for the survey recruitment: each focus group participant was asked to provide three email addresses of peers who would be willing to participate in our study.

#### Description of the Survey

The online questionnaire was designed in conjunction with the focus groups to obtain more detailed information of adolescents’ eating and buying behavior and their perceptions of *The Healthy School Canteen Program*. Tables 2 and 3 give an overview of the quantitative questions and the different answer types (single response categorical, check all that apply, or a rating scale) used for this paper.^2^ We chose to use 10-point scales for ratings as this has the advantage of being a very familiar format for students (34). The survey was programmed in Perseus Survey Solutions 6 (Perseus) and hosted at the Radboud University webserver.

**Table 2:** Dutch adolescents’ (*N* = 133) survey responses related to their eating and buying behaviors.

**A. Food consumption patterns and perceived healthiness of snacks**		
1. How many days per week do you have breakfast? [S]	Never or less than 1 day/week	6%
	1–6 days/week	30%
	7 days/week	64%

2. How many days per week do you have lunch? [S]	Never or less than 1 day/week	0%
	1–6 days/week	42%
	7 days/week	58%

3. How many snacks do you usually eat per day? [S] (e.g., biscuits, cake, chocolate, candy, chips, and fries)	Never or less than 1 per day	8%
	One per day	21%
	Two per day	41%
	Three per day or more	30%

4. How many glasses of sugar sweetened beverages do you usually have per day? (note: diet soft drinks are excluded here) [S]	None	24%
	Less than one per day	31%
	One per day	11%
	Two per day	13%
	Three per day or more	20%

5. How healthy are your snacks? [R]	Mean (SD)	5.7 (1.9)

**B. School break activities**		

1. I spend the school coffee/tea and lunch breaks usually… [S]	Alone or at home	2%
	With other students	98%

2. I spend the school coffee/tea and lunch breaks usually at [S]	The school canteen	17%
	Other areas	84%

3. During the school coffee/tea and lunch breaks I usually [C]	Chat with other students	92%
	Eat my sandwiches or snacks	85%
	Listen to music	19%
	Check social media	64%
	Play games	24%
	Do sports	5%
	Chill/relax with friends	74%

4. How important is the school break for you? [R]	Mean (SD)	8.2 (1.5)

**C. Eating and buying behavior during school time**		

1. What I eat at school is usually [S]	Brought from home	85%
	Bought at the school canteen	7%
	Bought elsewhere (e.g., supermarket)	5%
	Combination of above sources	4%

2. How many times per week do you buy food or drinks from your school canteen? [S]	Never	38%
	Once per week or less	42%
	Twice per week	12%
	Three times per week	4%
	Four times per week or more	5%

3. How much money do you spend per week on food or drinks from your school canteen? [S]	0 Euros per week	44%
	0–2 Euros per week	37%
	2–5 Euros per week	14%
	5 Euros per week or more	5%

4. How many times per week do you buy food or drinks from a vending machine at school? [S]	Never	50%
	Once per week or less	36%
	Twice per week	8%
	Three times per week	4%
	Four times per week or more	2%

5. How much money do you spend per week on food or drinks from a vending machine at school? [S]	0 Euros per week	58%
	0–2 Euros per week	36%
	2–5 Euros per week	6%
	5 Euros per week or more	0%

6. How many times per week do you buy food or drinks from outside the school area (e.g., supermarket and bakery)? [S]	Never	16%
	Once per week or less	47%
	Twice per week	21%
	Three times per week	8%
	Four times per week or more	8%

7. How much money do you spend per week on buying food or drinks outside the school area (e.g., supermarket and bakery)? [S]	0 Euros per week	21%
	0–2 Euros per week	41%
	2–5 Euros per week	26%
	5 Euros per week or more	11%

*R = rating 1 out of 10; C = check all that apply; S = single category response*.

*Percentages may not total 100% due to rounding*.

**Table 3:** Dutch adolescents’ (*N* = 133) survey responses on school canteen matters.

**D. School canteen as food provider**
1. What score would you give your school canteen? [R]	Mean (SD)	6.0 (1.5)
2. How important is the school canteen to you? [R]	Mean (SD)	4.9 (2.4)
3. What score would you give the food and drinks assortment of your school canteen? [R]	Mean (SD)	5.7 (2.0)
4. What is your perceived healthiness of the food and drinks assortment of your school canteen? [R]	Mean (SD)	5.4 (2.1)
5. What is your perceived unhealthiness of the food and drinks assortment of your school canteen? [R]	Mean (SD)	5.5 (2.2)
6. How important is it to you that your school canteen has a healthy food and drinks assortment? [R]	Mean (SD)	6.0 (2.5)

**E. Adolescents’ views on the Healthy School Canteen Program**		

1. How do you rate the *Healthy School Canteen Program* initiative? [R]	Mean (SD)	6.9 (2.6)
2. How important are the following aspects of the assortment in a healthy school canteen to you? [R]		
The products: taste good	Mean (SD)	8.1 (1.8)
Are healthy	Mean (SD)	6.5 (2.0)
Are cheap	Mean (SD)	7.8 (2.0)
Are fresh	Mean (SD)	7.7 (1.8)
Look appealing	Mean (SD)	7.1 (2.0)
Are high energy dense	Mean (SD)	5.7 (2.3)
Are low energy dense	Mean (SD)	5.1 (2.4)
Are hot	Mean (SD)	6.6 (2.1)
Are cold	Mean (SD)	6.0 (2.2)
The canteen’s offering has product variety	Mean (SD)	7.7 (1.7)
The canteen’s offering changes regularly	Mean (SD)	7.2 (2.1)

**F. Student involvement with the Healthy School Canteen Program**		

1. Does your school involve students in the organization of the school canteen, for example, by helping in the kitchen or at the register, or with the composition of the assortment of the canteen? [S]	Yes	12%
	No	32%
	Sometimes	20%
	I do not know	35%

**F. Student involvement with the Healthy School Canteen Program**		

2. In general, do you think it is important that students are involved with the organization of a healthy school canteen? [S]	Yes	64%
	No	36%
3. Suppose that students will be involved with the organization of a healthy school canteen. Which tasks would be most appropriate for them? [S]	Compose assortment	36%
	Create new products	36%
	Design of the canteen	9%
	Assist in sales	15%
	Advertising for the canteen	1%
	Other	3%
4. Would you like to be involved with setting up or organizing a healthy school canteen yourself? [S]	Yes	32%
	No	68%

*R = rating 1 out of 10; S = single category response*.

*Percentages may not total 100% due to rounding*.

The following topics were covered in the survey:

- *Food consumption patterns and perceived healthiness of snacks consumed*. Three questions were included about usual breakfast, lunch, and snack consumption patterns and one rating about the perceived healthiness of their snacks (anchored “not at all healthy” to “extremely healthy”). We also asked respondents to indicate their favorite healthy and unhealthy snacks with two open-ended questions.
- *School break activities*. To get a better understanding of the situational context in which adolescents consumed their foods, we asked three questions about their activities during the school breaks and one rating question on how important they find their school breaks (anchored “not at all important” to “extremely important”).
- *Eating and buying behavior during school time*. Seven questions assessed eating and buying behaviors during school time.
- *School canteen as food provider*. Adolescents rated their views on their current school canteen and the importance of a healthy offering in school canteens in six questions (score 1 out of 10, or anchored “not at all….” to “extremely …,” see Table 3).
- *Adolescents’ views on the Healthy School Canteen Program*. The program was defined in the survey as follows: “All Dutch schools should have a healthy food offering by 2015. A healthy school canteen consists of 75% of healthy products from the Wheel of Five (such as fruits, sandwiches and salads)^3^ and 25% of other products, such as sweet and savory snacks.” Subsequently, adolescents gave an overall rating of this initiative and indicated the importance of different features of a healthy school canteen.
- *Student involvement with the Healthy School Canteen Program*. First adolescents were asked whether their school actually involved students in the organization of the school canteen. Then, they were asked whether they thought it is important that students are involved in the organization of a healthy foods and drinks assortment at school. After being asked to imagine that students were indeed involved in the program, they then needed to indicate with an forced choice question “how students could best be involved” (e.g., helping with sales or composing food and drinks assortment; multiple responses possible) and “whether they would like to be involved with setting up or organizing a healthy foods and drinks assortment at school.” Finally, respondents were asked two open-ended questions relating to what the school could do to get them (i.e., the respondents) involved with the *Healthy School Canteen Program* and what they could do themselves to promote student involvement with the *Healthy School Canteen Program*.

##### Sociodemographics

At the end of the survey, respondents were asked to fill out information on: gender, age, height, weight, and education level.

#### Statistical Analysis

We first performed descriptive analyses and compared responses between subgroups (gender, age, and education level) with Chi-Square tests, Independent *t*-Tests, and One-Way ANOVAs. Relevant relations between ordinal categorical variables were further explored with a Chi-Square Linear-by-Linear association test. Spearman correlations were used to investigate the relation between age and the different 10-point rating scales. SPSS software 23.0 was used for all analyses. *p*-Values below 0.05 were considered to be statistically significant.

## Results

### Focus Groups

The key themes identified from the focus groups were largely consistent across different subgroups and are described jointly below.

### Healthy Foods and Eating Behavior

#### Eating: Looking and Feeling Good

Most adolescents stated that healthy eating was important. This was primarily discussed in relation to perceived short-term benefits: “not getting fat,” “a clean skin without pimples,” “feeling fit,” and “performing better at sports.” To a lesser degree, it was mentioned that healthy eating was also important for “not getting ill in the future.”

Generally, adolescents did not feel a need to change their current eating patterns as long as they felt and looked healthy. Only if noticeable body changes occur, such as visible weight gain (mostly mentioned by girls) or a lower performance with sports (mostly mentioned by boys) they would change current eating behaviors.

#### Risk Perception of Unhealthy Eating

Despite dichotomizing foods in “healthy” and “unhealthy,” adolescents’ views on the frequency at which unhealthy foods can be consumed were clearly subjective and variable. There was a strong belief that occasionally eating something unhealthy is not influencing one’s health. Hence, adolescents’ risk perception of unhealthy eating was low.

Girl, lower education level: “This negative impact …does not really exist, does it? As if something terrible would happen after having an unhealthy lunch.”

Yet, adolescents made a clear distinction between “healthy foods” that provide certain benefits, which were typically fresh fruit and vegetables, and “unhealthy foods” (sausage rolls, chips, chocolates, etc.), which were the tasty foods.

### Influences on Food Choice

#### Parental Influence and Autonomy

The majority of focus group participants indicated that parents were the key actors that influenced their eating behaviors. Parents were not only responsible for household grocery shopping and cooking but also acted as role models for healthy eating. Adolescents generally respected this and thought it was their parents’ natural role.

Girl, intermediate education level: “…if I would decide everything on my own… I don’t think it would end well.”

At the same time, however, adolescents frequently talked about their own responsibility and independence when it comes to eating. Several adolescents stressed that they, themselves, ultimately decide on what and when to eat. Autonomy with regard to food and eating was perceived as important.

Boy, high education level: “when you get to the stage that you go to school…you are independent and you control a part of your own life…it is not like your parents should follow you every single step you take.”

#### Influence of the School and Peers

Compared with parents and peers, schools were deemed to have less influence on adolescents’ eating behavior, although they were certainly recognized as an important influencer. Adolescents thought that the role of the school was mainly to ensure the availability of attractive and affordable healthy foods. Providing information and education about healthy eating was mentioned infrequently and only supported “if it is during school hours.” The influence by peers was not so much openly discussed but clearly present as illustrated by the following quote.

Girl, lower education level: “if another student asks me to go outside the school area [to the supermarket red.], I usually go with them and then you do buy something. And that is usually unhealthy food.”

### School Canteen As Food Provider

#### Backup or Extra Food

Adolescents reported to bring their own lunch (e.g., sandwiches) to school at most of the days. For most adolescents, the school canteen was of minor importance and primarily visited to buy “something extra.” As a result, foods from the school canteen were mostly regarded as “a treat” and primarily bought for taste and not for health. However, adolescents did expect to have plenty of healthy options in their school canteens, particularly for those instances when they forget to bring their own lunch.

#### Relative Costs

Product pricing was mentioned consistently as the key area for improvement in school canteens. First, adolescents compared school canteen prices directly to supermarket prices. Since supermarkets were perceived to be less expensive, students preferred to buy their foods and drinks there, whenever the school allowed this. Second, adolescents indicated that the healthy foods were often more expensive than the unhealthy at their school. Given that unhealthy foods were often also regarded to be more palatable options with more value for money, this “pushed” the youngsters toward the unhealthy choice.

Girl, lower education level: “The canteen is pretty expensive…You can better go to the supermarket where you can get more for half of the money. For only 1.80 euros you can easily buy two sausage rolls. It is more and more filling too.”

#### Freedom of Choice

All adolescents desired the freedom to choose from a range of foods and drinks in their canteen. These products should be appealing and affordable at first. In their view, a negative thing their school could do is to remove all unhealthy food options from a school canteen, and only offer healthy foods.

Girl, higher education level: “…I don’t understand why they are pushing for schools to offer so many healthy foods…a student just has a certain mind-set and thinks: I am going to eat something unhealthy.”

### Student Involvement with the School Canteen

#### Lack of Involvement

Despite having a clear opinion about the food and drinks assortment in school canteens, adolescents did not see an active role for themselves in the organization of a healthy school canteen. None of the focus group participants was actually involved with school canteen matters.

Boy, higher education level: “I think it becomes a topic when others start talking about it and when you notice it bothers them greatly too. That’s the point you are going to act. At the moment… that’s not really the case.”

Adolescents explained their lack of involvement also by the fact that they always had alternative ways to get the foods they liked, for instance by buying them in the supermarket. Furthermore, they did not have major issues with the current food assortment; if this was the case, however, then they could imagine that students would be more involved or willing to participate.

#### Need for Involvement

Adolescents were generally positive about the *Healthy School Canteen Program* with a 75% healthy and 25% unhealthy food and drink ratio. Although they did not see an active role for themselves, they nevertheless thought that students should be involved in the organization of a healthy food and drinks assortment at school. In general, they thought that adolescents were indeed important stakeholders in this initiative. However, they were skeptical and wondered whether their voice would be heard and transformed into concrete actions by schools. In general, adolescents did not have very positive experiences with “student participation”. Concrete rewards, such as study points or financial compensation, were discussed as ways to make it more attractive to become involved:

Boy, lower education level: “If I can make some money with it…. yes then I would be interested, and also go to the school board.”

### Survey

The survey results on adolescents’ consumption patterns, their school break activities and eating and buying behavior during school time are displayed in Table 2. We only report differences between subgroups (age, gender, and education level) when these were statistically significant.

### Healthy Foods and Eating Behavior

#### Food Consumption Patterns and Perceived Healthiness of Snacks

Somewhat more than half of the respondents had breakfast (64%) and lunch on a daily basis (58%). Students from a lower education level were more likely to skip breakfast than students from other educational levels, χ^2^(4, 133) = 11.72, *p* < 0.05, while girls skipped lunch more often than boys, χ^2^(1, 133) = 5.55, *p* < 0.05. Half of the respondents (55%) indicated to have less than one sugar sweetened beverage a day, with boys drinking significantly more sodas than girls, χ^2^(1, 132) = 13.02, *p* < 0.001. Most respondents (71%) indicated to have more than two snacks per day. In general, adolescents rated their snacks not as particularly healthy or unhealthy. Adolescents reported fresh fruit varieties (e.g., apples, bananas, and strawberries) as preferred healthy snacks, while chocolate and chips were regarded as favorite unhealthy snacks.

#### School Break Activities

The majority of students did not spend their (lunch)break at the school canteen, but in other areas in/around school. Time was mainly spent with others while eating lunch or snacks. Higher educated students more frequently stated to eat lunch or snacks during their break, χ^2^(2, 133) = 6.36, *p* < 0.05 and games during the break were mainly played by boys, χ^2^(1, 133) = 13.08, *p* < 0.001. Those who played games were significantly younger, *t*(1,130) = 2.02, *p* < 0.05. School breaks were regarded as very important to all adolescents, and the importance increased with age, *F*(7,124) = 2.57, *p* < 0.05.

#### Eating and Buying Behavior during School Time

Most adolescents brought their food from home (85%) and indicated to buy foods and drinks at their canteen “never to once per week or less” (80%). Only 5% of respondents indicated to spend more than 5 Euros or more per week in their canteen. When adolescents spent money on foods or drinks, they spent it more often outside (i.e., at supermarket) than inside the school (i.e., canteen or vending machine). A few differences between subgroups exist: boys spent more money on foods and drinks from their school canteen, χ^2^(3, 133) = 10.01, *p* < 0.05, and vending machines χ^2^(1, 133) = 8.42, *p* < 0.05. They also bought more frequently from vending machines χ^2^(4, 133) = 10.98, *p* < 0.05 and outside the school area, χ^2^(4, 133) = 14.94, *p* < 0.01 than girls. Younger adolescents brought more foods from home, *F*(3,127) = 4.04, *p* < 0.01, and spent less money in their school canteen, *F*(3,128) = 3.13, *p* < 0.05, than older adolescents. In addition, an association was found that those from lower education levels visited their school canteen more frequently, χ^2^(1, 133) = 5.98, *p* < 0.05, and also spent more money here, χ^2^(1, 133) = 9.21, *p* < 0.01. Finally, it was found that students from the intermediate level (HAVO) used the vending machine more often than the low (VMBO) and high (VWO) education levels, χ^2^(8, 133) = 19.87, *p* < 0.05.

The survey results on adolescents’ views on and involvement in the *Healthy School Canteen Program* are displayed in Table 3.

#### School Canteen As Food Provider

Adolescents rated their school canteen with a 6.0 on a 10- point scale (SD = 1.5). The school canteen was not considered to be of much importance to adolescents, with an average score of 4.9 (SD = 2.4). Neutral scores were also given for foods and drinks assortment, which was regarded neither healthy nor unhealthy. A positive correlation between age and perceived healthiness was found here, with higher scores among older participants, *r* = 0.24, *p* < 0.01. Overall, the study population indicated it was only slightly important that the school canteen had a healthy assortment. However, girls thought it was much more important than boys *t*(1,115) = 4.84, *p* < 0.001.

#### Adolescents’ Views on the *Healthy School Canteen Program*

Overall, adolescents were mildly positive about *The Healthy School Canteen* Program. Girls, however, were more positive about this initiative than boys, *t*(1,131) = 2.51, *p* < 0.05. Factors that were rated as most important were as follows: products should taste good, cheap and fresh, and come with variety. Girls found the healthiness, freshness, appearance, and low caloric content of products significantly more important than boys [*t*(1,131) = 3.65, *p* < 0.001; *t*(1,131) = 2.40 *p* < 0.05; *t*(1,101) = 4.80, *p* < 0.001; and *t*(1,131) = 2.27, *p* < 0.05, respectively].

#### Student Involvement with the *Healthy School Canteen Program*

Only a small proportion of adolescents was consistently involved with their school canteen, and this percentage was even smaller for lower education groups, χ^2^(6, 133) = 14.39, *p* < 0.05. Yet, 64% of the respondents indicated that it was important that students are involved in the organization of *The Healthy School Canteen Program*. Composing the assortment and creating new products were rated as the most appropriate involvement activities. Higher education students were more in favor of helping with sales and the design of the canteen than lower education levels, χ^2^(10, 133) = 19.25, *p* < 0.05. In general, however, the majority (68%) did not see an active role in the *Healthy School Canteen Program* for themselves.

Respondents indicated that students could help their school mostly with the composition of the assortment, by coming up with suggestions and ideas for new products that could be offered. Furthermore, students could help with preparing foods (e.g., sandwiches) and picking up shifts (e.g., helping with sales). When asked what students would need to fulfill their role, respondents indicated that they needed someone who would really listen to their ideas and/or supervision from their school to help with the canteen. Following questions on what the school could do to increase student involvement; they indicated that schools should reward the commitment of the students who would help in the canteen with discounts or free products, money, or course credits. Furthermore, respondents indicated that schools should better educate their students about the importance of healthy eating and the potential role of the school canteen in this. Students themselves, on the other hand, could help with the promotion of *The Healthy School Canteen Program* by handing out leaflets, making posters, or giving presentations about healthy eating. To a lesser extent, respondents indicated that they could buy more healthy foods themselves and inspire and motivate others to do the same. In general, however, they expressed that a well-founded school-wide initiative would be needed that would take students’ input seriously and follows up on their ideas. “If everyone is involved from the school, it will be easier for other students to contribute as well.”

## Discussion

Using a mixed-method approach, this study aimed to gain a better understanding of adolescents’ food and health perceptions along with their eating and buying behaviors within the context of their school environment. In addition, we explored adolescents’ willingness to be involved in the organization of a healthy food and drinks assortment at school.

Most adolescents expressed a strong belief that occasionally eating something unhealthy would not be detrimental to one’s health. They did not feel a need to change their current eating patterns, up to the point that noticeable body changes would occur, such as visible weight gain (mostly mentioned by girls) or a lower performance with sports (mostly mentioned by boys). Thus, as long as they felt and looked healthy, adolescents did not feel any need to change their current eating behavior. These results are consistent with previous observations that healthy eating simply equals a healthy weight for many teenagers (29, 36). Furthermore, our results underscore previous findings that there is little concern about future consequences of unhealthy (eating) behavior by this age group (28, 37, 38). One way to stimulate healthy behavior is to increase behavioral intent [cf. (27)]. This can be done, for instance, by educating adolescents about the individual factors affecting nutrition and health behavior (39). It should be acknowledged, however, that a heavy focus on deliberative planned action with regard to food and eating might not be particularly effective, given that automatic processes, such as food and eating habits, have been shown to be the most important predictors of unhealthy snack intake [cf. (40)].

In line with previous research (41, 42), food choice autonomy appeared to be another important theme for adolescents. On many occasions participants expressed their desire for independent food decision making, particularly at school. They frequently mentioned their own responsibility and independence when it comes to eating and also stressed that they, themselves, ultimately decide on what and when to eat. While adolescents fully agreed that healthy foods should be widely available in school canteens, they also expressed that access to unhealthy foods should not be prohibited. Yet, respondents also indicated that their parents were the key influencers of their eating behavior. Parents were not only responsible for household grocery shopping and cooking but also acted as role models for healthy eating. It has been proposed that adolescent autonomy is a co-construction between parents and adolescents (41), whereby parents could monitor and control the environment within which adolescents are given independence and responsibility. We envision that a similar process could be applied by schools, which is based on active encouragement of healthy eating while taking care that adolescents could also make their own food-related decisions.

Furthermore, it was found that school breaks are valuable to secondary students due to its highly social component, including hanging out with friends while having lunch. Peer influence on food choice and intake typically occurs in these types of social settings (43, 44). Indeed, adolescents indicated that their peers, next to their parents, played a role in their eating behavior. As expressed by one of the focus group members; “*if another student asks me to go outside the school area, I usually go with them and then you do buy something. And that is usually unhealthy food*.” Particularly during adolescence, children spend a lot of time with their friends and have a higher need to belong to a group and to be accepted by peers (45). Within this context, group norm setting is a powerful mechanism in determining an individual’s behavior. Social learning theory (46) and the operation of social norms (47) could explain similarities in diet and eating behaviors among young people. Previous research has established that adolescents’ snack and soft drink consumption are highly associated with intake of their peers (48), particularly when there is a high snack food availability in the canteen and vending machines (49). Therefore, it would be worthwhile to explore how the social context can be leveraged further in school food interventions that aim to stimulate adolescents to eat healthily.

Adolescents also indicated that they mostly bought their foods or drinks from other food venders such as supermarkets close to school. Such food outlets are often a more attractive option for youngsters, and their presence around schools has been associated with increased junk food consumption and obesity (50, 51). Besides the fact that these shops offer a greater variety of foods, adolescents also indicated that they get lower prices and better value for money in these outlets compared with their school canteen. With this in mind, respondents felt that they were often “pushed” towards these outlets. If schools want to “compete” with this, then it is important that they consider their canteen within this competitive field and optimize their assortment with various healthy, affordable, and pleasurable food options. Considering that respondents rated their current school canteen only mildly positive, it is clear that there is enough room for improvement for schools to better suit their assortment to their students’ needs and desires.

One further issue that emerged was that adolescents were often skeptical about follow-up actions by the school. Based on their previous experiences, they often felt that their input is not valued and/or does not lead to actual changes in school policy. Therefore, it is recommended that greater efforts are made by schools on the implementation of their students’ ideas; “*If everyone is involved from the school, it will be easier for other students to contribute as well*.” This approach is in line with previous secondary school health interventions that successfully applied a three step approach (1) survey student experiences and views at local school level, (2) involve young people in decision making by an action group comprised from students and staff, and (3) appoint an external facilitator or “critical friend” to ensure students voices are heard and to monitor progress (52). We recommend formalizing these steps in healthy food school interventions, such as *The Healthy School Canteen Program*. What would be most important, however, is that schools have a process in place to ensure that adolescents are consistently involved and feel that their suggestions are valued. Consequently, adolescents may feel more encouraged to come up with ideas and suggestions to improve their canteen. A reward system might be another solution to better involve adolescents in the organization of a healthy foods and drinks assortment at school. Adolescents indicated that being rewarded with discounts or free products, money, or course credits would stimulate them to be more involved. This is consistent with the findings of a study conducted in Ireland, demonstrating that adolescents are more motivated to be involved in a healthy eating intervention when a reward based system is used (29). Indeed, it has been shown that rewards can be used to enhance time and performance on tasks that initially hold little enjoyment (53).

In-depth qualitative or mixed-methods studies that focus on adolescents’ eating and buying behavior at school canteens and their willingness to participate in healthy food choice interventions are limited. This study provides valuable additional information to those involved in the development of school-based obesity prevention programs. Nevertheless, our results should be interpreted with the study limitations in mind. Although we selected students from different personal backgrounds (age, gender, education level, and region), our study made use of both purposive and snowball sampling and therefore cannot be regarded as representative for The Netherlands. Also, care should be taken with directly translating the general findings to other settings, regions and more so other countries. Due to practical constraints, we largely relied on self-report measurement, which is known to be prone to bias, particularly with adolescents (54). Some of the focus group participants also participated in the survey, which may have further biased the results. In future studies, it would be very worthwhile to increase power by including more respondents, specifically with regard to the subgroups reported in this study, and to include objective behavioral measures to evaluate adolescents’ views and behaviors within the context of environmental interventions designed to promote a healthy food and drinks assortment at schools.

By understanding adolescents’ food and health perceptions along with the factors that influence their buying and eating behavior within the school context, we can begin to design more effective nutrition intervention models. Using this information may help to increase participatory involvement in behavioral change interventions, as this has been shown to lead to greater effects (55). Therefore, it is recommended that secondary school food policies and interventions should take their target group into account. These teenage consumers are typically in a phase of seeking food choice autonomy, independence, and self-responsibility. With the current rise in overweight and obesity among young people, it is crucial to create a healthy food environment in schools that facilitates students to choose healthy food products. Besides improving the health aspects of the foods offered, schools should not lose sight of the importance of product price, good taste, and variety to make the school canteen attractive to students and to be able to compete with other food outlets. In our view, student involvement is crucial to ensure the success of a healthy school canteen strategy, but given adolescents’ limited motivation to get involved this will not be easy. It will require commitment and targeted effort to include them as stakeholders. Our advice would be to provide them with recognition and/or rewards to praise their time and effort. But most importantly: listen to them seriously and follow-up on their ideas in concrete and visible actions.

## Ethics Statement

All participants gave their informed signed consent, in accordance with the Declaration of Helsinki, before they took part in the study. Furthermore, the study was in accordance with the ICC/ESOMAR International Code on Market, Opinion and Social Research and Data Analytics. Given the non-invasive procedure of the research, the study did not fall under the Law Medical Research on Human Subjects. As such, the research was not reviewed by a Medical Ethical Committee. We did not apply for ethical approval from the Ethics Committee of the Faculty of Social Sciences, Radboud University. This was not obligatory at the time the study was conducted (in 2014), although this now has been changed.

## Author Contributions

RH and HB were involved with conceptualization of the research, design, analyses, data interpretation, and writing up the manuscript. FM was involved with conceptualization of the research, design, and reviewing the draft manuscript. JL was involved with data interpretation and reviewing the draft manuscript. AH was involved with analyses, data interpretation, and writing up the manuscript. All the authors approved the final version of the manuscript, ensured the accuracy and integrity of the work, and agreed to be accountable for all aspects of the work.

## Conflict of Interest Statement

RH, HB, and AH declare that they received funding from The Netherlands Nutrition Centre for their contributions toward this paper. The authors declare that there were no other relationships or activities that could be construed as a potential conflict of interest.

## Footnotes

^1^In 2014, when this study was conducted, a healthy school canteen was defined as having 75% of the visible assortment in accordance with the Guidelines of The Netherlands Nutrition Centre.

^2^The actual questionnaire consisted of 53 items; 10 items were left out for the purpose of this article.

^3^The Guidelines of The Netherlands Nutrition Centre are based on the Wheel of Five; a food based guide based on the Dutch Dietary guidelines 2015 (35).
